# Long-term outcomes of patients with refractory gastroesophageal reflux disease following a minimally invasive endoscopic procedure: a prospective observational study

**DOI:** 10.1186/1471-230X-14-178

**Published:** 2014-10-10

**Authors:** Wei-Tao Liang, Zhong-Gao Wang, Feng Wang, Yue Yang, Zhi-Wei Hu, Jian-Jun Liu, Guang-Chang Zhu, Chao Zhang, Ji-Min Wu

**Affiliations:** Department of Gastroesophageal Reflux Disease, Second Artillery General Hospital of the Chinese People’s Liberation Army, 16 Xinwai Street, Xicheng District, Beijing, 100088 China; Department of Vascular Surgery, Xuan Wu Hospital, Capital Medical University, Beijing, China

**Keywords:** Gastroesophageal reflux disease (GERD), Stretta procedure, Radiofrequency delivery, Long-term outcomes

## Abstract

**Background:**

Gastroesophageal reflux disease (GERD) is the most common digestive disease, affecting one third of the world’s population. The minimally invasive endoscopic Stretta procedure is being increasingly used as an alternative strategy to manage refractory GERD. However, long-term benefits of this procedure have to be further evaluated in clinical settings. This prospective observational study was therefore conducted to evaluate the outcome of patients with refractory GERD 5 years after the Stretta procedure.

**Methods:**

A total of 152 patients with refractory GERD underwent the Stretta procedure in our department between April 2007 and September 2008. They were followed up for 5 years, during which the primary outcome measures including symptom scores of heartburn, regurgitation, chest pain, cough and asthma and the secondary outcome measures including proton pump inhibitor (PPI) use and patients’ satisfaction were analysed at 6, 12, 24, 36, 48 and 60 months respectively.

**Results:**

Of the 152 patients, 138 completed the designated 5-year follow-up and were included in the final analysis. At the end of the 5-year follow-up, the symptom scores of heartburn (2.47 ± 1.22 vs. 5.86 ± 1.52), regurgitation (2.23 ± 1.30 vs. 5.56 ± 1.65), chest pain (2.31 ± 0.76 vs. 4.79 ± 1.59), cough (3.14 ± 1.43 vs. 6.62 ± 1.73) and asthma (3.26 ± 1.53 vs. 6.83 ± 1.46) were all significantly decreased as compared with the corresponding values before the procedure (P < 0.001). After the Stretta procedure, 59 (42.8%) patients achieved complete PPI therapy independence and 104 (75.4%) patients were completely or partially satisfied with the GERD symptom control. Moreover, no severe complications were observed except for complaint of abdominal distention in 12 (8.7%) patients after the Stretta procedure.

**Conclusion:**

The Stretta procedure may achieve an effective and satisfactory long-term symptom control and considerably reduce the reliance on medication without significant adverse effects in adult patients with refractory GERD, thereby having profound clinical implications.

## Background

Gastroesophageal reflux disease (GERD) is the most common chronic digestive disorder in which the reflux of gastric contents into the esophagus through the incompetent lower esophageal sphincter (LES) causes troublesome symptoms and/or complications [1]. Affecting one third of the world’s population, GERD inflicts significant direct and indirect costs and loss of productivity [2]. Apart from the economic burden, GERD profoundly impacts the quality of life of the affected individuals [3]. Currently, PPI-based pharmacological therapy in combination with lifestyle modifications (e.g., bed head elevation during sleep, changes in dietary habits, restriction on alcohol consumption, and body weight control) remains the mainstay of GERD management [4, 5]. As a class of potent gastric acid secretion-inhibiting agents, PPIs are effective in most GERD patients. However, PPI therapy involves an indefinitely prolonged or lifetime daily drug administration, which is associated with significant adverse effects [6]. Moreover, up to 40% of GERD patients are refractory to PPIs [7, 8]. These intrinsic drawbacks of PPI therapy necessitate the development of alternative strategies to manage GERD.

With extensive efforts worldwide, several laparoscopic and endoscopic modalities including various fundoplication techniques and the Stretta procedure that aim to correct the mechanical/physical defect underlying the gastric content reflux have been invented in the past few decades [9]. Although laparoscopic fundoplication, the 180-degree laparoscopic anterior fundoplication in particular [10], may achieve a satisfactory symptom relief and oesophagitis healing without significant complications in selected individuals [11] and is now regarded as the “gold standard” surgical management for refractory GERD [12], the wider application of this approach is limited by its invasiveness, high cost and inherent postoperative complications [13]. Accordingly, the Stretta procedure has been increasingly used as the first-line therapy for selected patients with PPI-refractory GERD [2, 14], since its approval by the Food and Drug Administration of the United States in 2000 [15].

The Stretta procedure is a minimally invasive endoscopic procedure that physically corrects the defect underlying the gastric content reflux while irrigating the overlying mucosa to prevent heat injury with radiofrequency energy delivered to the oesophageal wall and LES complex through a flexible catheter with a balloon-basket assembly and nickel-titanium needle electrodes [16]. The efficacy, safety, and patients’ satisfaction of the Stretta procedure have been evaluated in numerous prospective and meta-analysis studies, mixed results have been obtained [16–23]. This prospective observational study was therefore conducted to evaluate long-term results of the Stretta procedure in selective adult patients with PPI-refractory GERD.

## Methods

### Ethical statement

This prospective observational study was approved by the Institutional Review Board of the PLA’s Second Artillery General Hospital in Beijing and conducted in compliance with the ethical principles for medical research involving human subjects stated in the World Medical Association Declaration of Helsinki. Informed consent was obtained from all subjects.

### Subjects

A total of 152 patients with GERD, seeking care in the Department of Gastroesophageal Reflux Disease in the PLA’s Second Artillery General Hospital, Beijing, China, were recruited consecutively between April, 2007 and September, 2008. The inclusion criteria were: 1) GERD diagnosed by endoscopically evidenced esophagitis or abnormal oesophageal pH or DeMeester score ≥14.7 with symptom correlation ≥50%, and/or reflux episodes >73 during a 24 h ambulatory impedance monitoring; 2) lower than normal LES pressure detected by oesophageal manometry; 3) endoscopically confirmed Los Angeles grade A or B esophagitis; 4) non-hiatal hernia or small (<2 cm) hiatal hernia; 5) absence of Barrett’s oesophagus; 6) significantly impaired oesophageal motility; 7) persistent symptoms despite daily use of PPIs; and 8) age ≥18 years. Patients who fulfilled the criteria were stratified as extraesophageal symptoms and nonextraesophageal symptoms. Patients with contradictions for surgery, severe dysphagia, previous esophagogastric surgery, autoimmune disease, collagen vascular disease, and/or coagulation disorders were excluded.

### Treatment

Stretta radiofrequency was performed on all patients as previously described [24]. Briefly, the patient was sedated, and the distance to the gastroesophageal junction was measured under a gastroscope. Then the endoscope was withdrawn and a radiofrequency-delivering catheter, consisting of a flexible balloon-basket with four electrode needle sheaths, was introduced orally using a guide wire. The balloon was inflated 2 cm proximal to the squamo-columnar junction, the electrode needles were deployed, and radiofrequency energy was delivered for 1 min. The needles were then withdrawn, the balloon was deflated and the catheter was rotated 45°. These steps were serially repeated every 0.5 cm inwards, covering an area 2 cm above and 0.5 cm below the squamo-columnar junction.

### Outcome assessment

The primary outcome measures of this study were the frequency and severity of the major GERD symptoms including heartburn, regurgitation, chest pain, cough and asthma. Data on these outcome measures were collected through a standardized questionnaire using the 6-point Likert scale system. More specifically, the frequency was graded as 0 (none), 1 (less than once per week), 2 (once or twice per week), 3 (three or four times per week), 4 (five or six times per week) and 5 (more than six times per week); the severity was graded as 0 (none), 1 (slight), 2 (mild), 3 (moderate), 4 (severe) and 5 (extremely severe). The total of the frequency score and the severity score for each of these measures was designated as the symptom score.

The secondary outcome measures were medication independence and patient satisfaction following the Stretta procedure. Data on these measures were collected through a questionnaire survey consisting of 2 questions: 1) are you completely independent of PPIs; and 2) how are you satisfied with the treatment (not all/partially/fully).

The questionnaires were prepared in simplified Chinese and administered to the subjects before the Stretta procedure and at 6, 12, 24, 36, 48 and 60-months post-treatment on medications, respectively.

### Statistical analysis

Data were expressed as mean ± standard deviation (SD) unless specified otherwise. They were analysed by student t test or nonparametric test based on their nature. The statistical analysis software, SPSS-17.0 (SPSS Inc., Chicago, IL, USA), was used. Differences were considered significant when P < 0.05.

## Results

**Figure 1:**
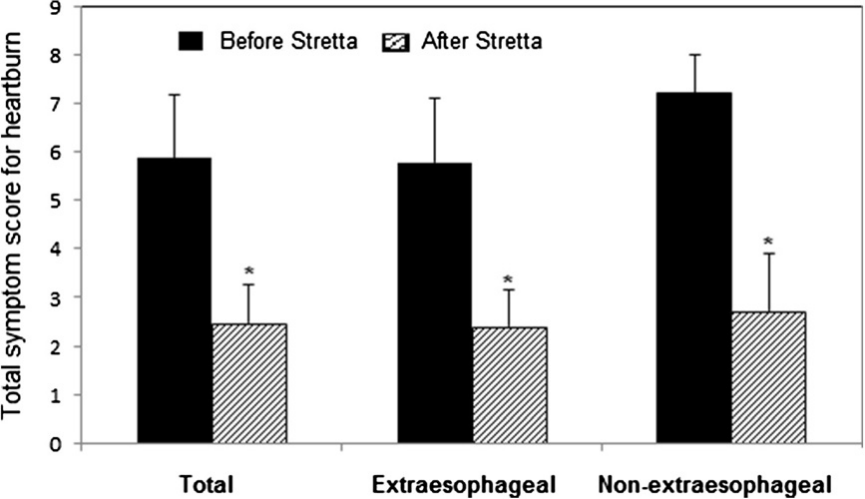
**Total heartburn score (frequency score + severity score) in 94 patients with or without extraesophageal symptoms before the Stretta procedure and at the 5-year follow-up.** A single asterisk (*) denotes a significant difference between bars in the same cluster (P < 0.001).

**Figure 2:**
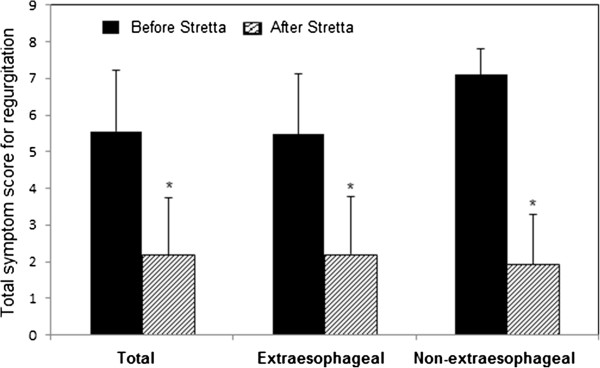
**Total regurgitation score (frequency score + severity score) in 82 patients with or without extraesophageal symptoms before the Stretta procedure and at the 5-year follow-up.** A single asterisk (*) denotes a significant difference between bars in the same cluster (P < 0.001).

**Figure 3:**
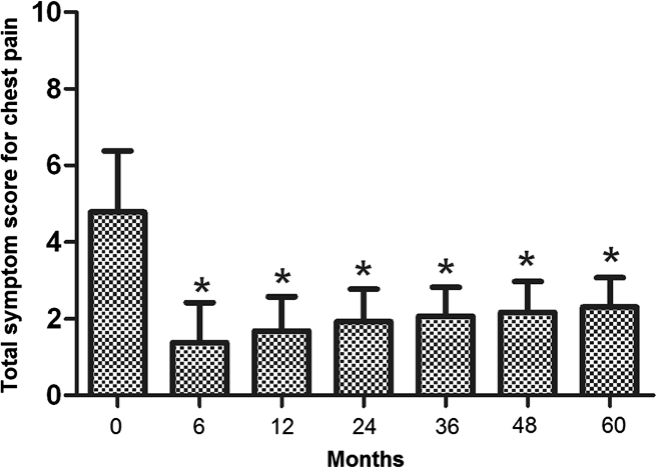
**Total chest pain score (frequency score + severity score) in 29 patients at the indicated time points.** A single asterisk (*) denotes a significant difference between bars in the same cluster (P < 0.001).

**Figure 4:**
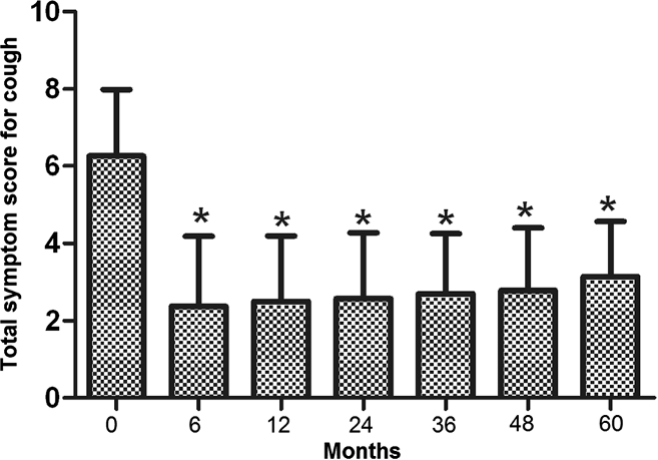
**Total GERD-related cough score (frequency score + severity score) in 75 patients at the indicated time points.** A single asterisk (*) denotes a significant difference between bars in the same cluster (P < 0.001).

**Figure 5:**
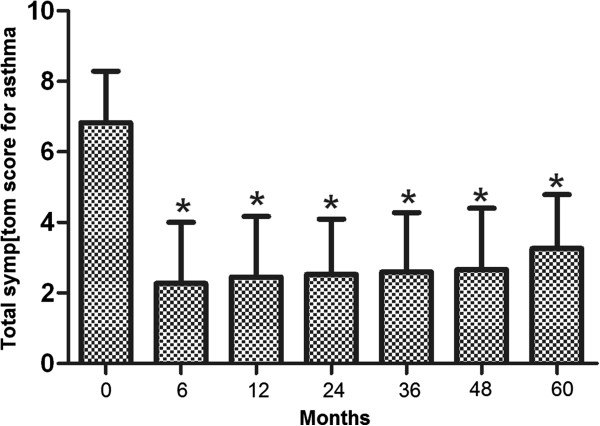
**Total GERD-related asthma score (frequency score + severity score) in 57 patients at the indicated time points.** A single asterisk (*) denotes a significant difference between bars in the same cluster (P < 0.001).

Initially, 152 patients entered the study. During the designated 5-year follow-up, 12 patients dropped of the study and 2 died of causes other than the Stretta procedure-associated complications. As a result, 138 (91%) patients had the complete follow-up data and were included in the final analysis. Of these patients, 87 had extraesophageal symptoms and 51 had no extraesophageal symptoms. There was no significant difference either in the average age (52.1 ± 13.0 vs. 52.1 ± 12.7, P = 0.94) or in male to female ratio (35/87 vs. 24/51, P = 0.43) between extraesophageal and non-extraesophageal symptom groups.In the 138 patients included in the analysis, 94 (68.1%) suffered from heartburn. The average total symptom score for heartburn was 5.86 ± 1.52 prior to the Stretta procedure, which was significantly reduced at 6 months after the Stretta procedure and the reduction sustained at 24, 38 and 48 months (data not shown). By the end of the 5-year follow-up, the total heartburn score was significantly lower as compared with the score before the Stretta procedure (P < 0.001) and the effect of the procedure was of no significant difference in patients with and without extraesophageal symptoms (P > 0.05, Figure 1). Eighty- two (59.4%) patients reported regurgitation, 29 (21%) chest pain, 75 (54.3%) cough and 57 (41.3%) asthma. The average total symptom score before treatment was 5.56 ± 1.65 for regurgitation, 4.79 ± 1.59 for chest pain, 6.62 ± 1.73 for GERD-related cough and 6.83 ± 1.46 for GERD-related asthma. In a pattern exactly the same as that for heartburn, the total symptom scores for regurgitation (Figure 2), chest pain(Figure 3), cough (Figure 4) and asthma (Figure 5) were all significantly reduced after the Stretta procedure (P < 0.001).

**Figure 6:**
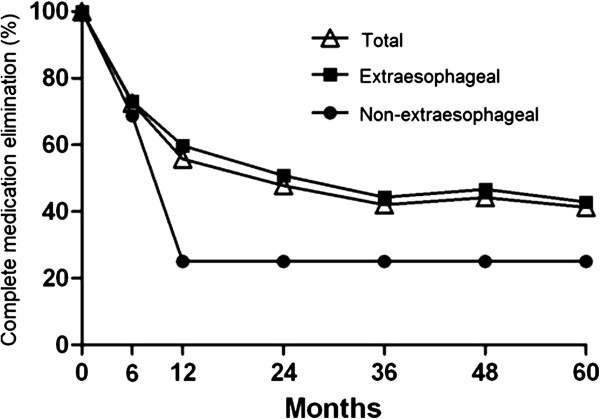
**Changes in the proportion of patients with (n = 87) and without (n = 51) extraesophageal symptoms who were independent of PPI therapy at the indicated time points.**

With regard to the secondary outcome measures, 38 (27.5%) of the 138 patients were completely off PPIs at 6 months following the Stretta procedure. By 60 months, 59 (42.8%) patients were completely off PPIs (Figure 6). In the 46 patients who were using PPIs as needed complained of extraesophageal symptoms before treatment. Medication was completely eliminated in 74.5% of the patients without extraesophageal symptoms at 60 months. Out of the 138 patients, 104 (75.4%) patients were fully or partially satisfied with the treatment and 94 (69.6%) expressed willingness to do the procedure again if necessary. In the 34 (24.6%) patients who were not satisfied with the treatment, 26 would definitely not undergo the procedure again.

During the 5-year follow-up, no post procedure perforations, mucosal laceration, bleeding episodes requiring transfusion, or deaths were observed. After the procedure, 12 patients complained of abdominal distention, which was moderately alleviated after treatment with trimebutine maleate tablets in combination with lifestyle modifications.

## Discussion

GERD significantly impairs the quality of life of the affected individuals. The current management strategies include lifestyle modifications, pharmacological therapies, surgical fundoplication, and, more recently, endoscopic procedures [9, 14]. An ideal GERD management modality should be able to effectively control symptoms, heal the injured oesophageal mucosa, and prevent complications in a convenient and inexpensive fashion. Both PPI-based pharmacological and surgical therapies fail to fall into the “ideal” treatment category PPI therapy is associated with a high refraction rate, troublesome daily administration and significant adverse effects while surgical fundoplication involves invasiveness and is associated with high cost and high risks of postoperative complications. Recently, the minimally invasive Stretta procedure has become an acceptable option for patients who are PPI-refractory and poor surgical candidates yet still require intensive treatment to adequately manage their GERD. Nevertheless, the long-term benefits of this minimally invasive procedure need to be further evaluated.

In this prospective study, we followed up 138 adult patients with PPI-refractory GERD for 5 years after the Stretta procedure. Our results clearly demonstrated that the Stretta procedure was effective to reduce the frequency and severity of GERD-associated symptoms (Figures 1, 2, 3, 4 and 5) including heartburn, regurgitation, chest pain, cough and asthma. The effect was evident at 6 months after the procedure and sustained over the entire observational period of 5 years. In the current literature, numerous studies have evaluated the efficacy of the Stretta procedure in the management of medication-refractory GERD [17, 22, 23, 25, 26], but few studies have followed up the patients for more than 4 years. Our long-term observations support the claim that the endoluminal Stretta procedure is an effective modality of symptom control in selected GERD patients [2].

In addition to the primary outcome measures discussed above, we also assessed the medication elimination post-treatment, the treatment-associated complications and patient satisfaction of the treatment. Prior to the Stretta procedure, 100% patients enrolled in the study used PPI. After the procedure, the medication elimination rate increased with time, from 27.5% at 6 months to 42.8% at 5 years (Figure 6). In two previous studies, complete PPI elimination was achieved in 75% and 86.4% of the GERD patients at 48 months after the Stretta procedure respectively [20, 21], which was slightly higher than the rate observed in our study. The discrepancy might be partially a result of differences in the quality of the drugs from different manufacturers. Nevertheless, this needs to be further determined in comparative studies in the future. Out of the 138 patients, 75.4% were partially or fully satisfied with the Stretta radiofrequency treatment. This satisfaction rate was very similar to that reported in a previous study where 77.0% of 558 patients from 33 institutions with a mean follow-up period of 8 months were satisfied with the Stretta procedure) [27]. In this study, no significant postprocedure complications were observed except for abdominal distention in 12 (8.7%) patients, supporting previous results on the safety of the Stretta procedure [20, 23].

Extraesophageal symptoms such as cough and throat clearing are not commonly associated with GERD [28]. However, the vast majority of the patients assessed in this study reported extraesophageal symptoms. Different from previous studies [20, 22, 25, 29], we performed stratified analyses of heartburn, regurgitation and PPI elimination based on the presence or absence of extraesophageal manifestations.We found that the Stretta procedure was effective to control heartburn and regurgitation very similarly in GERD patients with and without extraesophageal symptoms (Figures 1 and 2) but was less effective in eliminating PPI usage in patients with extraesophageal symptoms than in patients without extraesophageal symptoms (Figure 6). To our knowledge, little is known in the current literature regarding the efficacy of the Stretta procedure in GERD patients with and without extrasophageal symptoms, which warrants further investigations.

The mechanism underlying the therapeutic effect of the Stretta procedure remains to be further elucidated. It has been documented that endoluminal radiofrequency energy delivery to the gastroesophageal junction causes local inflammation, subsequent collagen deposition and muscular thickening in the LES, thus preventing acid reflux from the stomach [30, 31]. Alternatively, the Stretta procedure may achieve its clinical benefits through increasing the intragastric pressure needed to induce reflux and improve the gastric emptying [32–34] and/or decreasing compliance of the gastro-esophageal junction [17]. Moreover, the Stretta procedure may also exert its effect through the involvement of the vagal nerve, which remains controversial [9, 35].

## Conclusions

In summary, through prospective observation for 5 years, we demonstrated that the Stretta procedure was capable of controlling GERD symptoms effectively and safely. While a slight difference in the complete PPI elimination after the Stretta procedure was observed between GERD medication-refractory patients with and without extraesophageal manifestations, the effect of the procedure on changes in heartburn, regurgitation, chest pain, cough and asthma was not associated with extraesophageal manifestations. Our observations suggest that the Stretta procedure should be considered as a reliable treatment modality for adult patients with medication-refractory GERD.

## Authors’ information

ZG Wang (He himself was a so-called severe asthma patient with severe and intolerable respiratory symptoms requiring medication all the time for several years. After GERD was confirmed and fundoplication performed, asthma disappeared completely without applying any medication afterwards. Then he decided to devote to rescue those patients who suffered the same symptoms like him. Therefore, the GER Center established since 2006 in China), the pioneer of GERD-related airway disease research and practice in China, the founder of Center for GER of the Second Artillery General Hospital, Professor and Director of Vascular Institute of Xuan Wu Hospital of Capital Medical University, Lifetime President of Chinese Vascular Society, Vice President of International Society of Vascular Surgery.

## Abbreviations

GERD: Gastroesophageal reflux disease
PPI: Proton pump inhibitor
LES: Lower esophageal sphincter
SD: Standard deviation.
